# Supplementary material for: Secondary Treatment for Men with Localized Prostate Cancer: A Pooled Analysis of PRIAS and ERSPC-Rotterdam Data within the PIONEER Data Platform
Source: J Pers Med. 2022 May 5;12(5):751. doi: 10.3390/jpm12050751 (PMC9146310; doi:10.3390/jpm12050751)
Supplement: Supplementary file 1 [file jpm-12-00751-s001.zip › jpm-1693195-supplementary.pdf]

Supplementary Figure S1: Cumulative Incidence of RP

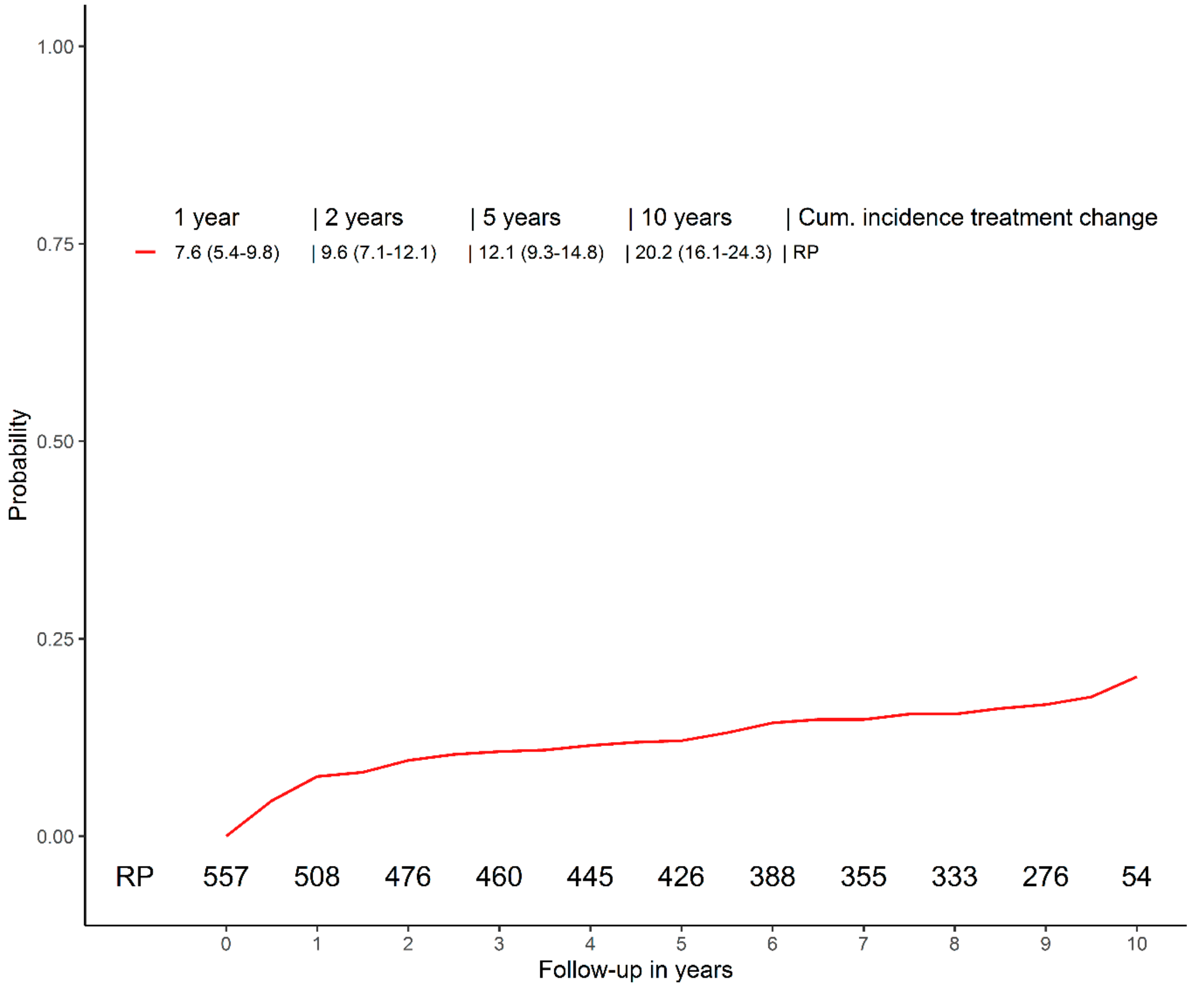

Supplementary Figure S2: Cumulative Incidence of RT

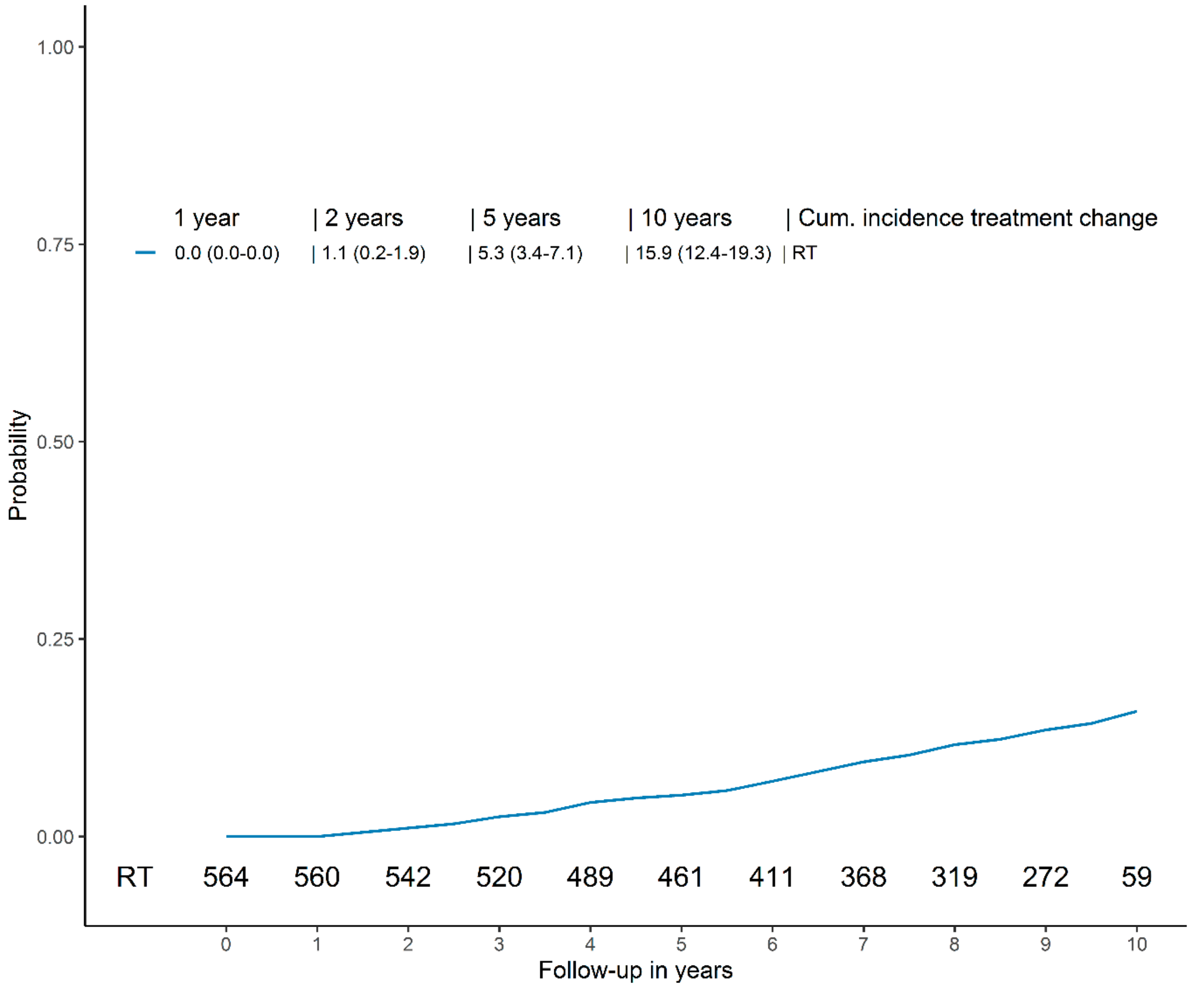

Supplementary Figure S3: Cumulative Incidence of AS

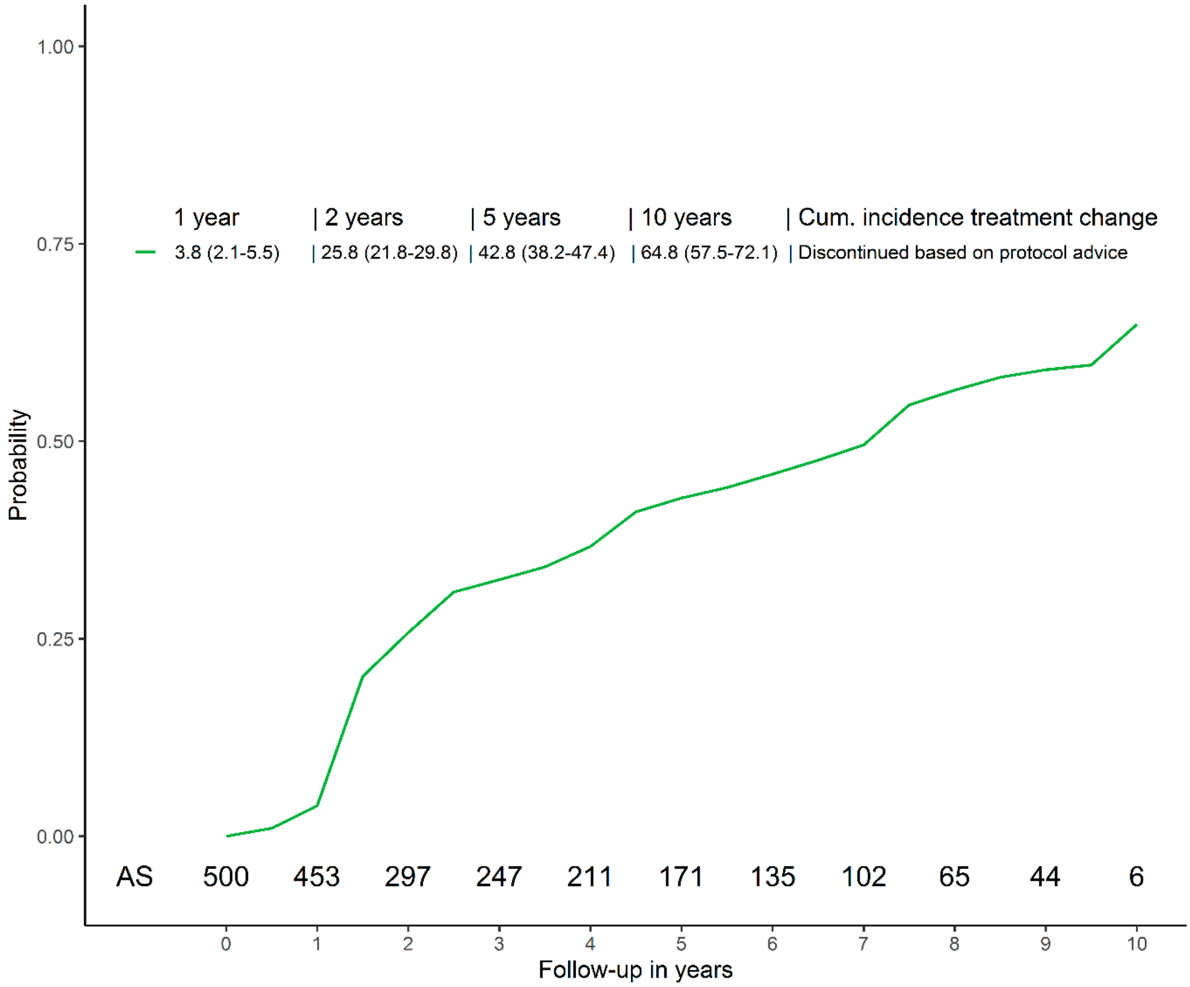

Supplementary Figure S4: Cumulative Incidence of AS after discontinuation of AS

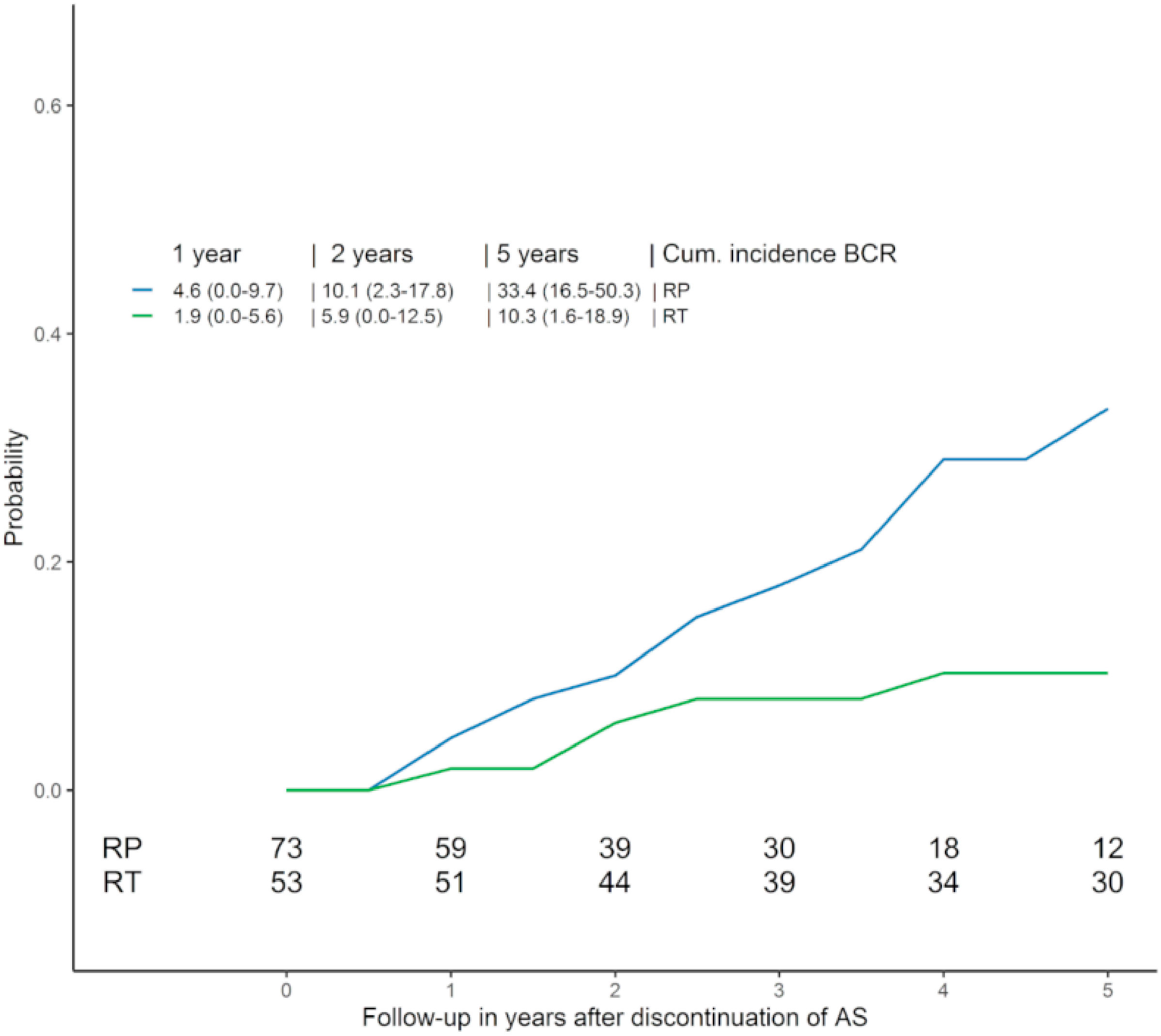

PIONEER

E. Smith; J. N'Dow; K. Plass; M. Ribal; N. Mottet; R. Shepherd; L. Moris; M. Lardas; T. Van den Broeck; P-P M. Willemse; N. Fossati; K. H. Pang; R. Campi; I. Greco; M. Gacci; S. Serni; A. Bjartell; S. Evans-Axelsson; R. Lonnerbro; A. Briganti; D. Crosti; M. Meoni; R. Garzonio; G. Gandaglia; C. Bangma; M. Roobol; S. Remmers; D. Tilki; A. Auvinen; T. Murtola; T. Visakorpi; K. Talala; T. Tammela; A. Siltari; M. Van Hemelrijck; K. Beyer; S. Lejeune; S. Caputova; S. Byrne; L. Fialho; A. Cardone; P. Gono; M. De Vetter; I. Panagiotopoulou; F. Ugolini; A. Rodriguez; B. De Meulder; C. Auffray; I.-A. Balaur; N. Taibi; A. Thouvenin; A. Hijazy; S. Power; N. Zounemat Kermani; K. van Bochove; M. Moinat; M. Kalafati; C. Bernini; D. Horgan; L. Fullwood; M. Holtorf; D. Lancet; G. Bernstein; M. I. Omar; S. MacLennan; S. MacLennan; S. Tripathee; M. Wirth; M. Froehner; B. Brenner; A. Borkowetz; C. Thomas; F. Horn; K. Reiche; M. Kreuz; A. Josefsson; D. Gasi Tandefelt; J. Hugosson; J. Schalken; H. Huisman; T. Hofmarcher; P. Lindgren; E. Andersson; A. Fridhammar; A. Asiimwe; F. Verholen; J. Zong; J.-E. Butler-Ransohoff; T. Williamson; K. Chandrawansa; R. Waldeck; A. Bruno; R. Herrera; E. Nevedomskaya; S. Fatoba; N. Constantinovici; A. Mohamed; C. Steinbeißer; S. Kedhagae; M. Maass; P. Torremante; S. Evans-Axelsson; E. Dochy; F. Pisa; M. D. Voss; Z. Devecseri; T. Abbott; A. Kiran; C. Dau; K. Papineni; J. Wang-Silvanto; R. Snijder; V. Doyé; X. Wang; A. Garnham; M. Lambrecht; R. Wolfinger; S. Rogiers; L. Antoni; A. Servan; K. Pascoe; P. Robinson; B. Jaton; D. Bakkard; H. Turunen; O. Kilkku; P. Pohjanjousi; O. Voima; L. Nevalaita; K. Punakivi; C. Reich; S. Ratwani; E. Longden-Chapman; D. Burke; M. Licour; M. Ang; S. Payne; A. Yong; F. Lujan; S. Le Mare; J. Hendrich; B. Franks; M. Bussmann; I. Köhler; G. Juckeland; D. Kotik.

ERSPC Rotterdam study group

W.J. Kirkels; J.B.W Rietbergen, I.W. van der Cruijsen, R. Raaijmakers, S.H. de Vries, S. Roemeling, C. Gosselaar, T. Wolters, R.C.N. van den Bergh, P.J. van Leeuwen, M. Bul, X. Zhu, H.A. van Vugt , L.P. Bokhorst, A.R. Alberts, F-J. Drost, J.F.M. Verbeek, D.F Osses, H. Luiting, S. Remmers, R. Hogenhout, J.W. Salman, L.D.F. Venderbos, G. Yurdakul, A. Boeken-Kruger, C. Wijburg, M. Forouzanfor, M. de Boer, R. Postma, A.N. Vis, R. Hoedemaeker, G.J.L.H. van Leenders, B. Blijenberg, P.J. van der Maas, S. Otto, G. Draisma, P. Beemsterboer, M. Essink-Bot, I. Korfage, R. Boer, M. Wildhagen, W. Merkelbach, W. Hoekstra, J. Blom, R.A.M. Damhuis, A. Reedijk, R. Kranse, D.W. Roobol, W. Roobol, E. van den Berg, G-J de Zwart, C.G.A.M. Franken-Raab, M. van Slooten-Midderig, A. Smit, V. van der Drift, E. de Bilde, L. Mani, M. Visser-van Dongen, H. Versteeg-Leenheer, B. Zoutendijk, N. Vink, H. van Meurs, A. E. de Bruijn.

PRIAS

F.M. Bentvelsen; J. Jaspars; E. Planken; A. Bendiksby; Olav Andreas Hopland; V. Berge; T. Habuchi; R.M. Potjer; R. Raaijmakers; W.M. Stomps; J.J. Vis; P.A. Wertheimer; A.G.M. Zeegers; G. Smits; P.J. van den Broeke; D. van der Schoot; H. Jansen; J. van Brakel; K. Zaccai; M. Tijnagel; I. van Onna; E.H.G.M. Oomens; P.J. Posthumus; M. Schuit; E. van Muilekom; H.G. van der Poel; A. Kahnamelli; A. Briede; H. Lau; T. Pickles; C. Rikken; I. Cordia; M. Kummeling; R. van den Bergh; D. Duijvesz; D.M. Somford; H. Vergunst; E.L. Koldewijn; P.E.F. Stijns; W. Scheepens; A. Villers; J. Fonseca; O. Capoun; S. Sakamoto; R. Gregoir; B. Guillonneau; K. van Dalen; R. Spermon; A. Jungwirth; W. Boellaard; J. Boormans; M.B. Busstra; B. Weening; C.H. Bangma; F.H. Drost; S. van den Heuvel; K. de Mooij; W.J. Kirkels; L.P. Bokhorst; M. Bul; M. van Mierlo; P.C.M.S. Verhagen; M.J. Roobol; W. Roobol; N. Azevedo; F. Sanguedolce; P.P. Perez; M. Maffezzini; M. Kortleve; A. Viddeleer; M. Eto; K. Suzuki; M.

Kiewiet de Jonge; F. Froeling; T. Sugiyama Shiga; C. Arsov; D. Caasen-Findeisen; R. Rabenalt; H. Krueger; J. Schmitges; J. Teishima; S. Maruyama; C.A. Becerra; P.F. Martinez; W. Villamil; J. M. Caballero Giné; R. Bastus; E.R. Barselo; M. Montesino; A.S. Rannikko; B. Rikken; W. Obara; H.P. Beerlage; J.R. Oddens; R.A. Schipper; H. Sasaki; Y. Kakehi; H. Kinoshita; A. Gayer; M. Schiffer; J. Winkle; T.A. Ozkan; N. Schmeller; S. Aaltomaa; A. Terai; M. Multanen Kyorin; O. Ukimura; T. Inoue; A. Yokomizo; C.D. Vera Donoso; J.M. Alapont-Alacreu; M. Martinez-Sarmiento; C. de Castro; M. Roeleveld; R. Pelger; A.M. Reicherz; K.H. Tully; N. Atassi Stroelin; R. Sosnowski; M. Schotman; J. Verlind; T. Roeleveld; S.D. Bos; E. Alleman; M. Asselman; B. Santerse; H. Leenknegt; M. Pit; S. Khoe; N. Hendolin; N. Terada; T. Hakariya; H. Okuno; L.W. Tim; Y. Kitamura; Y. Tomita; M. Nakayama; M. Uemura; P. Hellström; F. Staerman; G. Rodemer; C.F. Ng; P. Chiu; C. Nitz; S. Laabs; N. Bosch; S. Saito; Y. Sakai; H. Kitamura; T. Tsukamoto; M. Leskinen; K. Hashine; A. Paganelli; A. Lapini; E. Trenti; M. Roscigno; M. Tanello; F. Badenchini; T. Magnani; M.F. Alvisi; T. Rancati; L. Pasini; M. Gallucci; O. Caffo; G. Cicchetti; G. Conti; R. Sanseverino; P. Ditonno; G. Martorana; V. Altieri; A. Geboers; A. Noordzij; S. Tenbreul; A. Sonneveld; P.L.M. Vijverberg; I. van den Berg; J. Blom; E.R. Boevé; R. Nooter; J. Rietbergen; S. de Vries; I. van den Berg; H. Wilkens; J.W. Langeveld; A. E.Treiyer; T. Anagnostou; M. Gillich; J. Hanske; T. Tammela; Y. Naya; E. Bruijnes; E. Hoogendijk; F. van der Windt; B. Zeijlemaker; K. Kawashima; N. Kamiya; K. Mitsuka; T. Nomoto; T. Fukumori; K. Akakura; Y. Maeda; B. Wijsman; I. van Oort; C. Hoeks; R. Lazarov; P. Papavassilis; A. Semjonow; W. Oosterlinck; N. Mottet; F.G. Veiga; A. Bjartell; J.S. Uribe; H. Fukuhara; M. Nakamura; T. Keul; J. Trunk; C. Hawke; M. Rice; M. Rice; D. Gyomber; N. Lawrentschuk; L. Dodds; L. Johns-Putra; H. Fan Chan; F. Macneil; M. Louie-Johnsun; P. Anderson; K. Rantall; P. Kearns; P. Ruljancich; A. Jayathillake; C. Chemasle; Q. King; S. La Bianca; D. Murphy; M.J. Monsour; A. Vega Vega; C. Smiles; D. Malouf; J. Grummet; M. Patel; V. Chalasani; N. Awad; P. Rashid; S. Brough; A. Tan; M. de Bruin-Titulaer; A. N. Vis; I. Hara; M. Leter; N. Tsuchiya; H. Matsuyama; H. Matsumoto; Soe Fung Kon Jin; A.Q.H.J. Niemer; E.B. Cornel; G. Molijn; S.P. Stomps; J. Beck; S. van Selm; T. Willems; P. de Vries; R. Bosshardt.
